# CCR9 in cancer: oncogenic role and therapeutic targeting

**DOI:** 10.1186/s13045-016-0236-7

**Published:** 2016-02-16

**Authors:** Zhenbo Tu, Ruijing Xiao, Jie Xiong, Kingsley M. Tembo, Xinzhou Deng, Meng Xiong, Pan Liu, Meng Wang, Qiuping Zhang

**Affiliations:** Department of Immunology, School of Basic Medical Science, Wuhan University, Wuhan, 430071 China

**Keywords:** CCR9, CCL25, Biomarker, Chemoresistance, Metastasis, Targeting therapy

## Abstract

Cancer is currently one of the leading causes of death worldwide and is one of the most challenging major public health problems. The main challenges faced by clinicians in the management and treatment of cancer mainly arise from difficulties in early diagnosis and the emergence of tumor chemoresistance and metastasis. The structures of chemokine receptor 9 (CCR9) and its specific ligand chemokine ligand 25 (CCL25) have been elucidated, and, interestingly, a number of studies have demonstrated that CCR9 is a potential tumor biomarker in diagnosis and therapy, as it has been found to be highly expressed in a wide range of cancers. This expression pattern suggests that CCR9 may participate in many important biological activities involved in cancer progression. Researchers have shown that CCR9 that has been activated by its specific ligand CCL25 can interact with many signaling pathways, especially those involved in tumor chemoresistance and metastasis. This review, therefore, focuses on CCR9 induction activity and summarizes what is currently known regarding its role in cancers and its potential application in tumor-targeted therapy.

## Background

Chemokines are a class of small proteins with molecular weights of approximately 8–14 kDa that, once combined with their homologous receptors, can activate and chemoattract leukocytes. Chemokine receptors are G protein-coupled receptors with seven transmembrane domains. Chemokines and their receptors play vital roles in the migration of thymocytes and maturation in normal and inflammatory environments. At the same time, they have great potential for use in tumor-targeted therapy [1, 2] in which, cancer-related chemokines could promote tumor proliferation, angiogenesis, and chemoresistance [3–5]. In the past decade, chemokine ligand 25 (CCL25)/chemokine receptor 9 (CCR9) have been found in a wide variety of tumors and have been associated with tumor chemoresistance and metastasis. This review will examine the expression of CCR9 in cancer, recent insights into the mechanisms of CCL25/CCR9 that are involved in tumor chemoresistance and metastasis, and the potential application of CCR9-based targeted therapy.

## Structure and characteristics

### CCR9

**Fig. 1:**
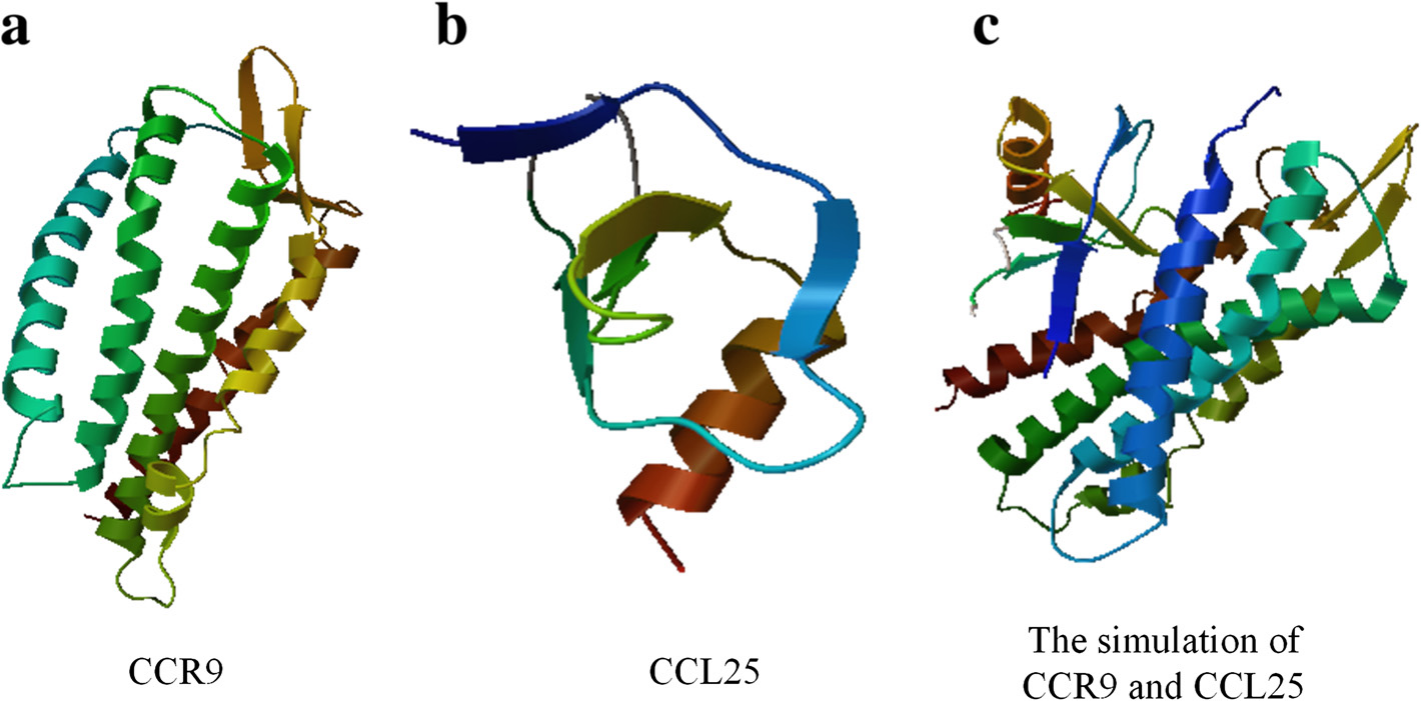
The structures of CCR9 and CCL25. The molecular structures of CCR9 (**a**) and CCL25 (**b**) from the UniProt web site (http://www.proteinmodelportal.org/query/uniprot). The simulated interaction of CCL25 and CCR9 was performed by MGLTools 1.5.6 software based on the structures of CCR9 and CCL25 (**c**)

CCR9 is located on chromosome 3p21.31 and belongs to the β-chemokine receptor family. It consists of transcripts A (369 amino acids, 42 kDa) and B (357 amino acids, 40.8 kDa). The molecular structure of CCR9 (44 to 241 amino acids) is shown in Fig. 1a. CCR9 is mainly distributed in immature T lymphocytes and on the surface of intestinal cells, and it plays a role in T lymphocyte development and tissue-specific homing when bound to its specific ligand.

### CCL25

CCL25, also known as thymus-expressed chemokine (TECK), belongs to the CC chemokine family and is the specific ligand for CCR9. CCL25 is located on chromosome 19p13.2 and consists of isotype 1 (150 amino acids, 16.6 kDa), isotype 2 (84 amino acids, 9.5 kDa), and isotype 3 (149 amino acids, 16.5 kDa). The molecular structure of CCL25 (28 to 93 amino acids) is shown in Fig. 1b. The simulated interaction between CCL25 and CCR9 is shown in Fig. 1c.

## The expression of CCR9 in cancer

Several studies have found that CCR9 is highly expressed in a variety of cancers (Table 1). These studies have confirmed that various factors lead to the upregulation of CCR9, such as TNF-α, which promotes CCR9 expression in human breast cancer MCF-7 cells [6], Notch signaling has been observed to induce CCR9 upregulation, which plays a vital role in T-lineage acute lymphoblastic leukemia (T-ALL) [7]. Moreover, we are finding that certain non-coding RNAs, such as miRNA and lncRNA, can mediate the expression of CCR9 and further affect its biological function.

**Table 1:** The expression of CCR9 in cancer patients

Tumors	Cell type	Methods	Expression	References
T-ALL	PBMCs (*n* = 21)	FCM	91.9 %	[25]
Melanoma	Circulating tumor cells (*n* = 21)	FCM	57 %	[58]
Melanoma	Primary specimens (*n* = 32)	IHC	69.7 %	[59]
Melanoma metastasis	Small intestinal metastases (*n* = 102)	QPCR	86 %	[30]
Mediastinal large B cell lymphomas	Lymphoid tissue (*n* = 22)	IHC	90.9 %^a^	[60]
Large B cell lymphoma	Gastric extranodal diffuse lymphoma (*n* = 10)	IHC	58.33 %	[61]
Large B cell lymphoma	Gastrointestinal lymphoma (*n* = 41)	IHC	66 %^b^	[62]
Ovarian cancer	Cancer tissues (*n* = 43)	IHC	Significantly higher	[11]
Lung cancer	Lung biopsies (*n* = 50)	WB	1.2^c^	[63]
Hepatocellular carcinoma	Cancer tissues (*n* = 240)	IHC	55.8 %	[9]
Breast cancer	Moderately differentiated cancer tissues (*n* = 18)	IHC	50 %	[64]
	Poorly differentiated cancer tissues (*n* = 18)	IHC	>75 %	
Colon cancer	Adenomatous foci (*n* = 46)	IHC	2.26 ± 0.06^d^	[65]
Nasopharyngeal carcinoma	Carcinoma tissues (*n* = 42)	IHC	80.95 %	[66]

*FCM* flow cytometry, *PBMC* peripheral blood mononuclear cell, *QPCR* quantitative polymerase chain reaction, *IHC* immunohistochemistry, *WB* western blot

^a^Proportion of positive tumor cells >50 % (20/22)

^b^3^+^ CCR9 staining

^c^Control tissue (*n* = 50), CCR9/β-actin <0.3

^d^Normal colon epithelium had a mean staining intensity of 1.60 ± 0.04 (*n* = 55)

## The pathway of CCL25/CCR9 in cancer

With recent statistics showing that one in four deaths in the USA is due to cancer [8]. Cancer mortality remains high, while the number of cancer survivors continues to increase. Some of the challenges faced by clinicians in the management and treatment of cancers include difficulties in diagnosing cancer in the early onset stages and the emergence of tumor chemoresistance and metastasis.

Recently, researchers have focused on CCR9 expression in selected cancers and on how CCR9 expression can be manipulated as a potential tumor biomarker in cancer diagnosis and treatment. The interaction of CCL25/CCR9 has been shown to activate many signaling pathways in cancer, especially those involved in tumor chemoresistance and metastasis. Therefore, in this review, we focus on CCR9 as a potential tumor biomarker and on how the CCL25/CCR9 pathway is involved in tumor chemoresistance and metastasis.

### CCR9 as a tumor biomarker

CCR9 expression has been shown to be increased in various cancers, and CCR9 is specifically expressed in certain tissues. Studies have revealed that ectopic expression of CCR9 may be mediated by the downregulation of p21 and p27 and upregulation of cyclin D1 to enhance cell proliferation and tumorigenicity in hepatocellular carcinoma cells. Additionally, the ectopic expression of CCR9 has been shown to be an independent prognostic factor for the overall survival of hepatocellular carcinoma patients [9]. Other researchers have shown that the upregulation of CCR9 was associated with a poor response to infliximab, which is an anti-tumor necrosis factor-α (TNF-α) antibody drug used in psoriasis patients. Therefore, CCR9 expression can act as a novel prognostic marker and therapeutic target for hepatocellular carcinoma [9] and may be a useful biological marker of the clinical efficacy of infliximab therapy in psoriasis patients [10].

### CCL25/CCR9 induces tumor chemoresistance

#### CCL25/CCR9 causes conformational change of PI3K dimers to activate PI3K/AKT pathway

The interaction of CCL25/CCR9 can cause conformational changes that allow PI3K dimers to activate PI3K. Activated PI3K on the plasma membrane can produce a second messenger (PIP3) that binds with the intracellular AKT PH domain and phosphoinositide-dependent kinase (PDK) 1 to phosphorylate AKT at Thr308 and PDK2 to phosphorylate AKT at Ser473. Activated AKT can activate the phosphorylation of NF-κB or mTOR signaling molecules, or it can inhibit its downstream target protein, GSK-3β, to mediate cancer proliferation and apoptosis [11–13]. Recent studies have shown that CCL25/CCR9, via activating the PI3K/AKT signaling pathway, mediated anti-apoptotic processes in lung cancer [12], induced etoposide resistance in prostate cancer [14], and induced cis-platinum resistance, which is dependent on PI3K and not FAK, in breast and ovarian cancer [15, 16].

#### CCL25/CCR9 enhances the interaction of P-gp and ERM to increase drug efflux

We found that CCL25/CCR9 involvement in the resistance of TNF-α-induced apoptosis in T-ALL depends on Livin, thus suggesting that CCL25/CCR9 plays an anti-apoptotic role [17]. Furthermore, we obtained a multi-resistant T-ALL cell line, MOLT4/DOX, which was derived from MOLT4 (which has a naturally high expression of CCR9) through doxorubicin dosing screening. Then, we investigated this multi-resistant cell line and found that CCR9 induces resistance to chemotherapy drugs, which could be blocked by CCR9 antibodies. We confirmed that CCL25/CCR9 influences the interaction of P-gp and the cytoskeleton protein ERM to increase P-gp efflux, thus mediating T-ALL tumor chemoresistance [18].

#### CCL25/CCR9 mediates STAT signaling to weaken cytotoxic effect

Tumor resistance to cytotoxic T lymphocytes mainly limits T cell tumor immunotherapy. Khandelwal and colleagues performed a siRNA rapid high-throughput screen and found that in T cells, STAT signaling pathways were mediated by CCR9 and affected Th1 cytokine secretion to weaken the cytotoxic effect. Therefore, inhibiting CCR9 expression in vivo could significantly improve the effect of tumor-specific T cell-mediated immune therapy [19].

#### CCL25/CCR9 activates β-catenin to induce tumor chemoresistance

**Fig. 2:**
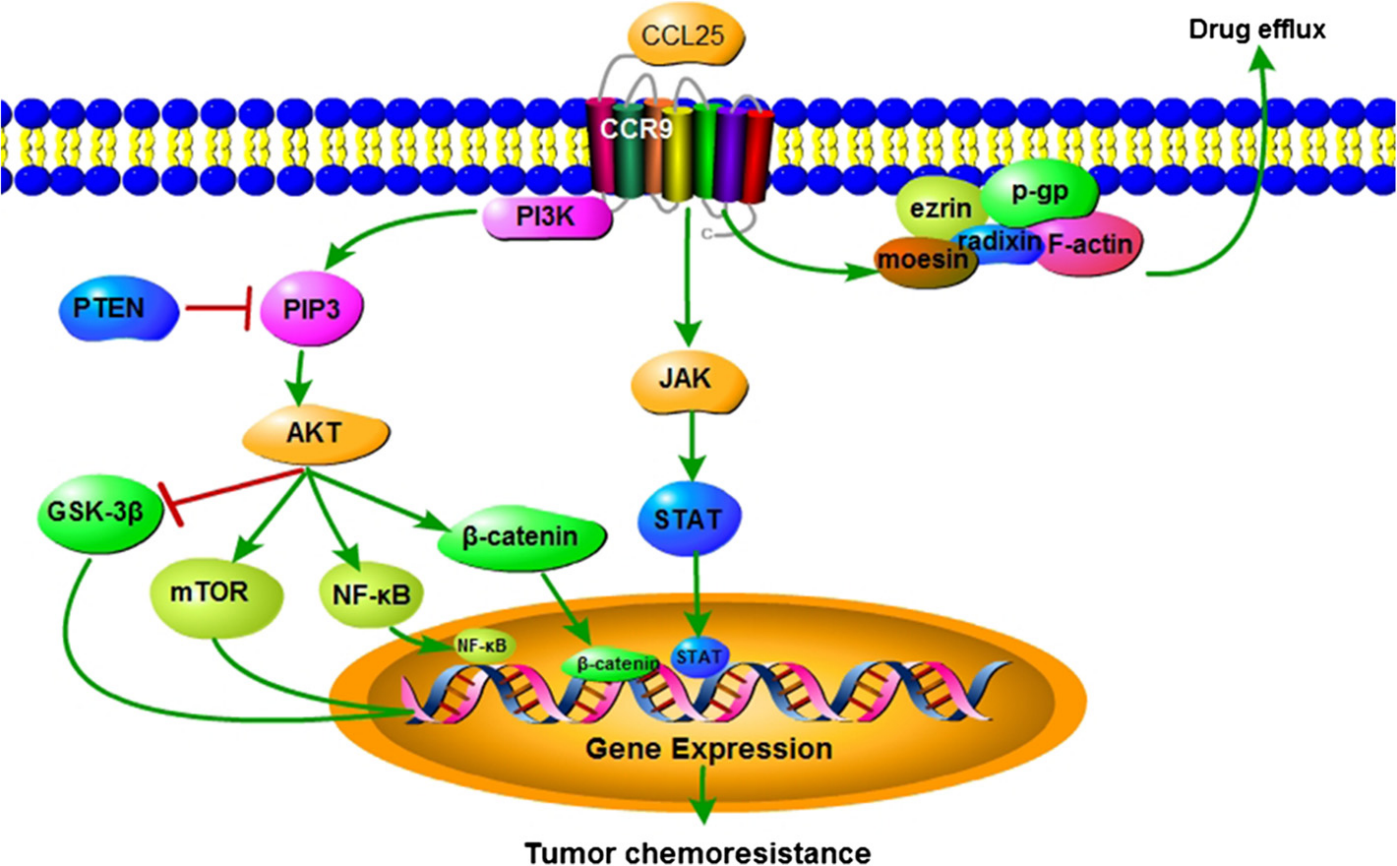
Diagram summarizing the reviewed mechanisms by which CCR9 induces different aspects of tumor chemoresistance. The interaction of CCL25/CCR9 can induce tumor chemoresistance via the PI3K-AKT-(GSK-3β/mTOR/NF-κB/β-catenin) and JAK-STAT pathways. The interactions between P-gp, ERM, and F-actin can induce tumor chemoresistance via the CCL25/CCR9 pathway

CCR9-mediated activation of β-catenin and the resulting downstream effects were effectively inhibited by blockade of the PI3K/AKT pathway. Further, β-catenin activation induced by CCR9 increased the expression of Cyclin E1, Cyclin D1, and E-Cadherin to increase the lethal dose of gemcitabine in pancreatic cancer, suggesting that CCR9/β-catenin signaling enhances pancreatic cancer chemoresistance [20]. A diagram of the mechanism of CCR9-mediated tumor chemoresistance is shown in Fig. 2.

### CCL25/CCR9 induces tumor metastasis

#### CCL25/CCR9 increases the expression of MMP2 and MMP9 to degrade collagen type IV

The degradation of the extracellular matrix and basilar membrane is a key step in the process of cancer cell invasion and metastasis that mainly involves matrix metalloproteinases (MMPs). MMPs are proteolytic enzymes that are closely associated with tumor metastasis. Most MMPs contain an affiliate area to adjust the catalytic activity and identify the substrate, while others have affiliate areas that have no catalytic activity but can play a role in tumor progression. For example, MMP-2 and MMP-9 have three fibronectins, which repeatedly inserted in the catalytic area to connect the substrates gelatin, collagen, and laminin. Therefore, MMP-2 and MMP-9 can degrade collagen type IV, which is a major component of the basilar membrane. Studies have found that the interaction of CCL25/CCR9 can increase the expression of MMP-2 and MMP-9, which induce tumor metastasis in a variety of cancers, such as ovarian cancer [21], prostate cancer [22], non-small cell lung cancer [23], and endometriosis [24].

#### CCL25/CCR9 induces cancer cells polarization and microvilli absorption

**Fig. 3:**
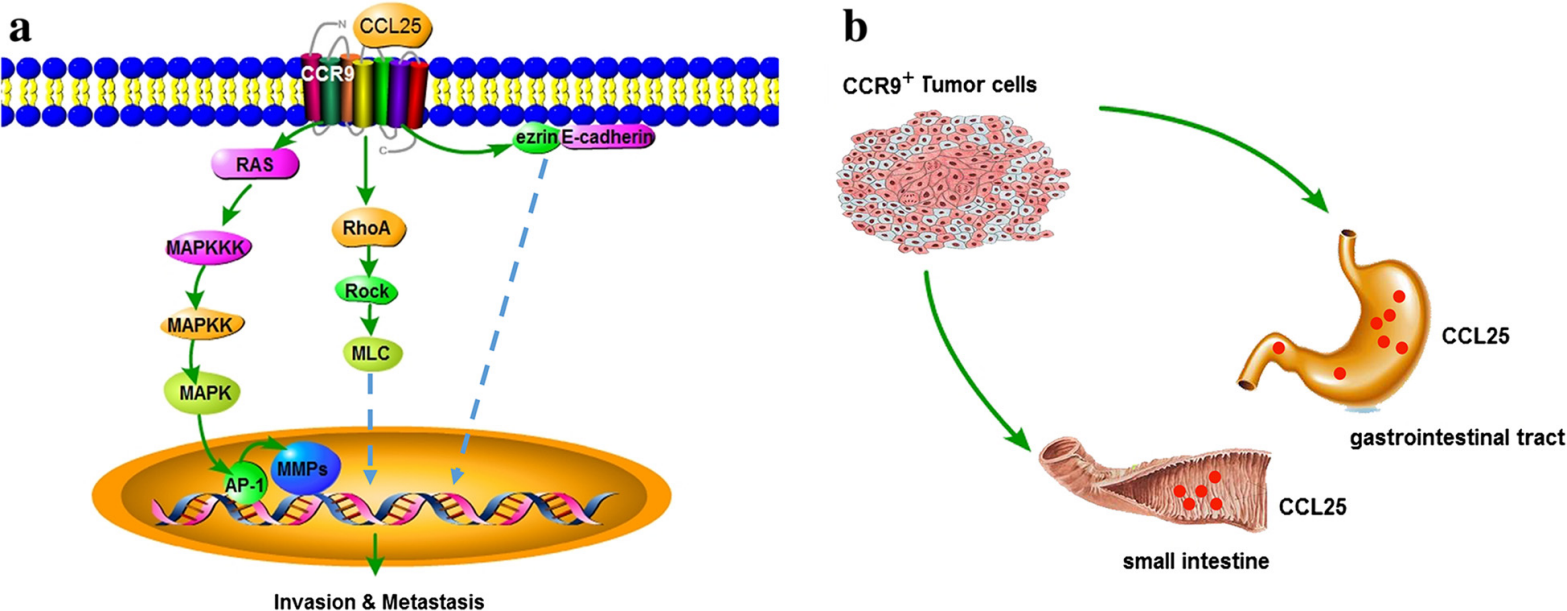
Diagram summarizing the reviewed mechanisms by which CCR9 induces different aspects of tumor metastasis. **a** The interaction of CCL25 and CCR9 can induce tumor metastasis via the RAS-MAPK-MMP pathway, the RhoA-Rock-MLC pathway, and ezrin signaling. **b** CCR9-high-expressing tumor cells are targeted to the small intestine and gastrointestinal tract by CCL25/CCR9 signaling. The *solid arrows* indicate that these signaling pathways have confirmed; the *dashed arrows* show that these signaling pathways need to be further validation

Our previous study found that CCR9 is highly expressed in CD4^+^ T cells from T-ALL patients, with little or no expression in normal T cells. The chemotaxis and adhesion effects on leukemia cells were ablated when CCR9 was internalized on the T-ALL CD4^+^ T cells, which suggests that CCR9 is closely related to the infiltration and metastasis of leukemia cells [25]. Further studies showed that CCL25/CCR9 induces MOLT4 cell polarization and microvilli absorption to participate in leukemia infiltration and trafficking via the RhoA-ROCK-MLC and ezrin pathway [26, 27]. A diagram of the mechanism of CCR9-mediated tumor metastasis is shown in Fig. 3.

#### Paracrine CCL25 induces tumor metastasis

Most cancer cells upregulate CCR9 to mediate metastasis, and some cancers secrete CCL25 in a paracrine fashion; these cells include pancreatic cancer PSCs and PANC-1 cells, which then induce metastasis by binding the secreted CCL25 to CCR9 receptors in the nearby tissues [28]. The mechanism of metastasis remains unclear.

#### CCR9 as a lymphocyte homing receptor

Chemokine receptors can also act as homing receptors for chemotaxis-specific cell homing to specific positions. Studies have shown that CCR9 is highly expressed in melanoma skin lesions [29], and that melanoma cells are specifically targeted to the small intestine when CCL25/CCR9 signaling is activated [30, 31]. In addition, CCR9 is highly expressed in adult lymphoblastic leukemia cells, and the CD4^+^ T cells that infiltrate the gastrointestinal tract show high CCR9 expression as detected by immunohistochemistry, suggesting that the infiltration of leukemia cells into the intestine is closely related to CCR9 expression in patients [32].

#### CCL25/CCR9 inhibits the bioactivity of PTEN gene

The expression of cytokines in the cancer microenvironment can affect the PTEN gene. It has been observed that CCR9-induced tumor proliferation and migration activity is increased with the loss of PTEN in T-ALL models, suggesting that PTEN loss can inhibit tumor metastasis [33]. Therefore, the mechanisms of CCL25/CCR9-induced tumor metastasis are complex and require further research.

**Fig. 4:**
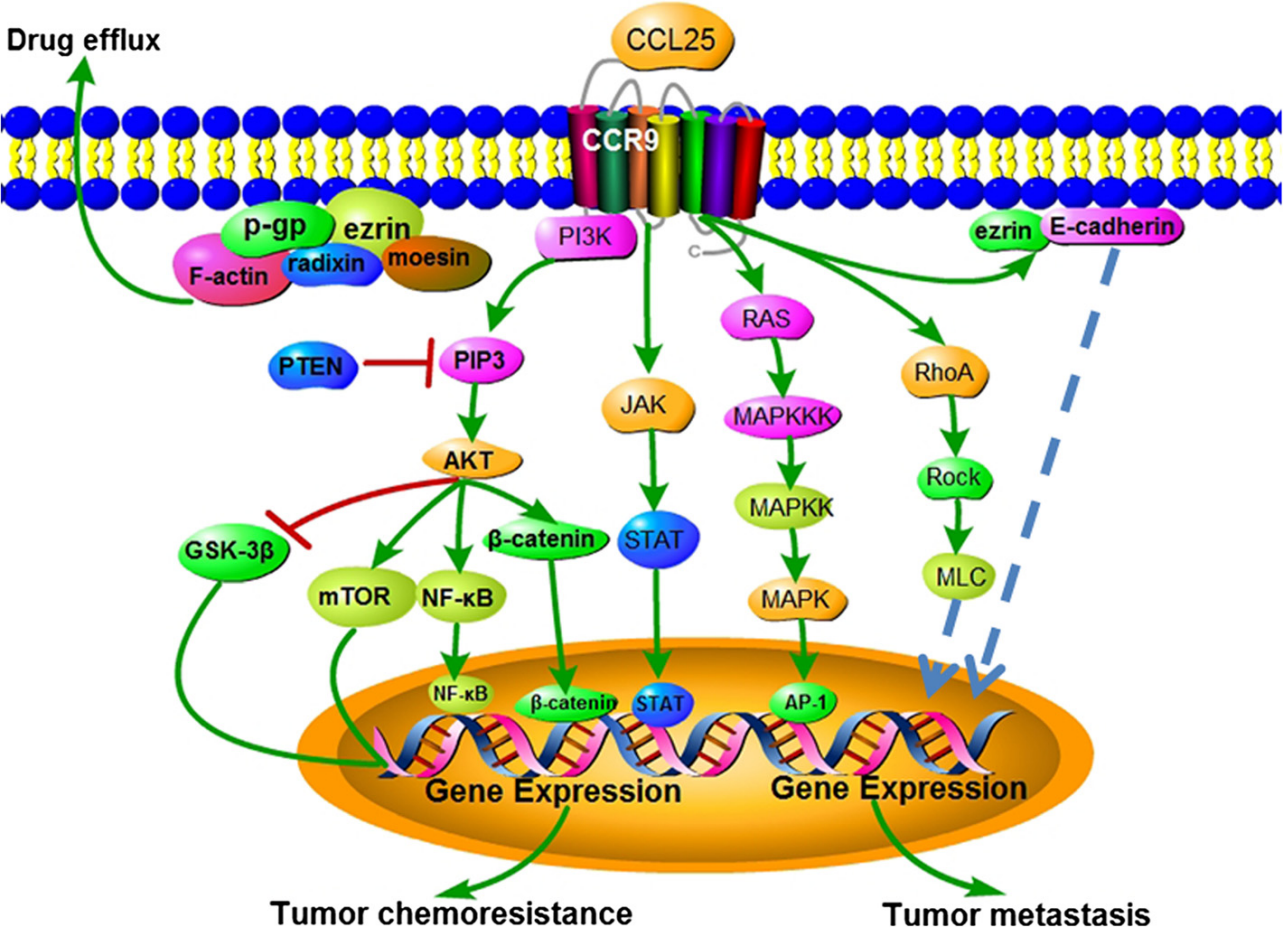
Diagram summarizing the reviewed mechanisms by which CCR9 induces different aspects of tumor chemoresistance and metastasis. The *solid arrows* indicate that these signaling pathways have confirmed; the *dashed arrows* show that these signaling pathways need to be further validation

The different signaling pathways of CCL25/CCR9 that are involved in cancer chemoresistance and metastasis are shown in Table 2, and a diagram of the mechanism of CCR9-mediated tumor chemoresistance and metastasis is shown in Fig. 4.

**Table 2:** The signaling pathway of CCL25/CCR9 in cancer chemoresistance and metastasis

	Pathway	Reference
Tumor chemoresistance	PI3K/AKT	Lung cancer [12], prostate cancer [14], breast cancer [15], ovarian cancer [16]
	P-gp/ERM/F-actin	T-ALL [17, 18]
	STAT	Breast cancer [19]
	β-catenin/cyclin	Pancreatic cancer [20]
Tumor metastasis	MMPs	Ovarian cancer [21], prostate cancer [22], non-small cell lung cancer [23], endometriosis [24]
	RhoA-ROCK-MLC/ezrin	T-ALL [26, 27]
	Paracrine of chemokines	Pancreatic cancer [28]
	Lymphocyte homing	Melanoma [29, 31], lymphoblastic leukemia [32]
	PTEN signaling	T-ALL [33]

## Targeted therapy research

When we identify a molecule that is highly expressed in cancer and rarely expressed in normal tissues, it is important to know whether there is clinical value to this information. Some researchers have developed an anti-CD19 monoclonal antibody with high killing activity against B cell malignancies, acute lymphoid leukemia, and B-ALL cells based on a specific B cell marker (CD19) [34–36]. Debra and colleagues found that anti-HuD-based immunotoxin therapy might be an effective alternative treatment for patients with small cell lung cancer and neuroblastoma because the HuD antigen is expressed in 100 % of small cell lung cancer cells and in over 50 % of neuroblastoma cells [37]. Presently, others and we found that CCR9 is highly expressed in various cancers (Table 1), and have focused on targeting therapy based on CCR9.

### CCR9 antibody

A number of studies have developed chemokine receptor-specific monoclonal antibodies as potential targeted therapies based on the important roles of chemokines and their receptors, such as CXCR4 [38], CXCR5 [39], CCR4 [40], and CCR7 [41] in cancer. Chamorro and colleagues identified and developed a mouse anti-human CCR9 IgG2b monoclonal antibody (91R) that can recognize an epitope within the CCR9 N-terminal domain and that inhibits the growth of subcutaneous xenografts in mice with an 85 % reduction in tumor size compared with controls. Tumor reduction was consistent, and apoptotic cells and tumor necrotic areas increased as the number of proliferating cells decreased in 91R-treated mice [42].

### Immunotoxin

Drug-targeted therapy is usually selective for specific systems, organs, tissues or cells, and this plays an important role in the area of the specific targeted treatment. Recently, targeted therapy has mainly included immunotoxins [43, 44], bispecific antibodies [45, 46], Nano-agents [47], ultrasound and microbubbles [48], and other specific inhibitors [49]. The CCR9-specific antibody has presented a challenge in terms of delivery to specific tissues due to its larger molecular weight, hence limiting its application. Most importantly, few antibodies are clinically useful as single agents because they do not efficiently kill cancer cells [50]. To further enhance the anti-tumor efficiency and reduce side effects, receptor-targeted therapy has been developed [51]. CCR9-based targeted therapy mainly involves an immunotoxin-CCL25 combination that has a small molecular weight and a high specificity. Its chemotactic ability to target cells makes it useful in the targeted therapy of CCR9^+^ malignant cancers with good prospects for application.

**Fig. 5:**
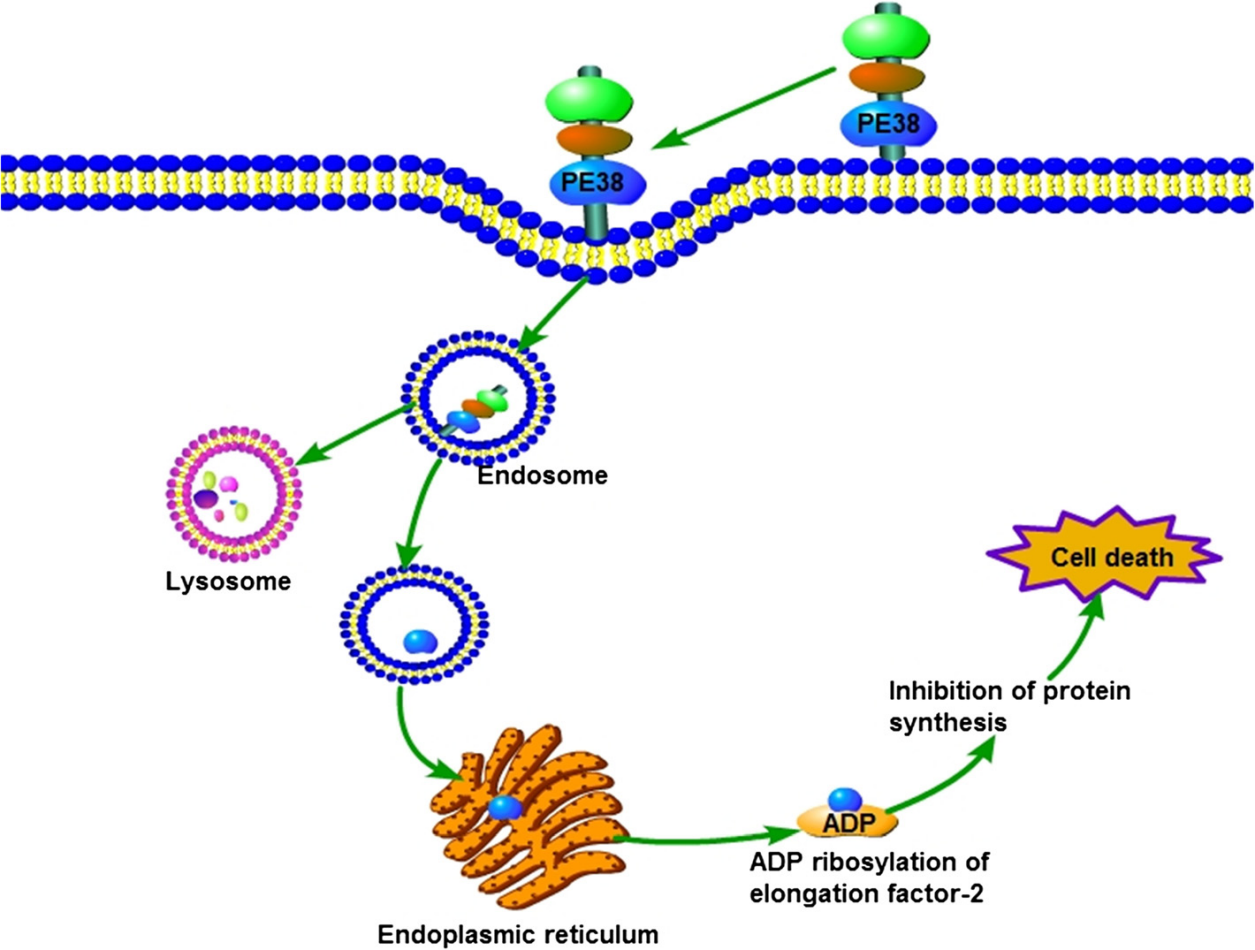
The mechanism of PE38-induced cell death. The immunotoxin is internalized when it binds with its specific receptor via the endolysosomal system to the Golgi, and it is further transported to the endoplasmic reticulum. Then, it can cause ADP ribosylation and elongation factor 2 inactivation to halt protein synthesis and eventually lead to cell death

We previously developed a CCL25-PE38 fusion protein using genetic engineering. PE38 is a derivative of Pseudomonas exotoxin A (PE). PE38 is composed of domains II and III of PE; domain II (278 to 389 bp) contains a furin cleavage site, while domain III (430 to 638 bp) has ADP-ribosylating activity [52]. When the ligand binds with its specific receptor, the immunotoxin is internalized via the endolysosomal system to the Golgi apparatus and is further transported to the endoplasmic reticulum, where PE38 is activated through reduction of a disulfide bond and cleavage by the protease furin at a site that separates the Fv from the catalytic fragment of PE38. Subsequently, the activated PE38 translocate to the cytosol, where it ADP-ribosylates and inactivates elongation factor 2, an essential component of the translation apparatus, thus halting protein synthesis and eventually leading to cell death [53]. The mechanism of PE38 action is shown in Fig. 5 [54].

Our results demonstrated that CCL25-PE38 is able to specifically kill MOLT4 cells via induction of apoptosis. This result suggests that CCL25-PE38 could suppress the growth of CCR9-positive cancers, as CCR9-high-expressing human T-ALL cells underwent apoptosis when exposed to a PE38 toxin fused to a CCL25 ligand. However, it can be said that it cannot completely eliminate the established cancer, as some parts of the expanding tumors do not express CCR9, thereby rendering CCL25-PE38 ineffective [55].

### Others

We also found that T-ALL cells can increase the expression of IL-4 after treatment with IL-2 and that IL-2 can activate the PKC and GRK2 signaling pathways to induce CCR9 internalization, thereby reducing leukemia cell infiltration and metastasis [56].

## Conclusions

CCL25/CCR9, as a member of the CC chemokines and their receptors, is known to be involved in chemotactic cells, lymphocyte development, survival, proliferation, and migration. Recently, researchers have found that CCL25/CCR9 plays an important role in tumorigenesis and is mainly involved in tumor chemoresistance and metastasis, which hamper the effects of conventional surgery, radiotherapy, and chemotherapy. Therefore, CCR9 has become a potential targeted molecule for cancer therapy because it is highly expressed in various cancers (Table 1). Targeting therapy drugs can be delivered into exact positions and can then be gradually released, thus reducing drug systemic side effects with enhanced antitumor efficiency [57]. Nevertheless, substantial work is still required, and future research efforts will provide us with a pharmacological basis of the therapeutic use of targeted therapy in cancer and a basis for the further investigation of other potential anti-CCR9 agents.

## Abbreviations

CCL25: chemokine ligand 25
CCR9: chemokine receptor 9
MMPs: matrix metalloproteinases
PDK: phosphoinositide dependent kinase
PE: Pseudomonas exotoxin A
T-ALL: T-lineage acute lymphoblastic leukemia
TECK: thymus-expressed chemokine
TNF-α: tumor necrosis factor-α
